# *PRDM10* directs *FLCN* expression in a novel disorder overlapping with Birt–Hogg–Dubé syndrome and familial lipomatosis

**DOI:** 10.1093/hmg/ddac288

**Published:** 2022-11-28

**Authors:** Irma van de Beek, Iris E Glykofridis, Jan C Oosterwijk, Peter C van den Akker, Gilles F H Diercks, Maria C Bolling, Quinten Waisfisz, Arjen R Mensenkamp, Jesper A Balk, Rob Zwart, Alex V Postma, Hanne E J Meijers-Heijboer, R Jeroen A van Moorselaar, Rob M F Wolthuis, Arjan C Houweling

**Affiliations:** Amsterdam UMC, Vrije Universiteit Amsterdam, Department of Human Genetics, De Boelelaan 1117, Amsterdam, The Netherlands; Amsterdam UMC, Vrije Universiteit Amsterdam, Department of Human Genetics and Cancer Center Amsterdam, De Boelelaan 1117, Amsterdam, The Netherlands; University of Groningen, University Medical Center Groningen, Department of Genetics, Hanzeplein 1, Groningen, The Netherlands; University of Groningen, University Medical Center Groningen, Department of Genetics, Hanzeplein 1, Groningen, The Netherlands; University of Groningen, University Medical Center Groningen, Department of Pathology, Hanzeplein 1, Groningen, The Netherlands; University of Groningen, University Medical Center Groningen, Department of Dermatology, Hanzeplein 1, Groningen, The Netherlands; Amsterdam UMC, Vrije Universiteit Amsterdam, Department of Human Genetics, De Boelelaan 1117, Amsterdam, The Netherlands; Radboudumc, Department of Human Genetics, Geert Grooteplein Zuid 10, Nijmegen, The Netherlands; Amsterdam UMC, Vrije Universiteit Amsterdam, Department of Human Genetics and Cancer Center Amsterdam, De Boelelaan 1117, Amsterdam, The Netherlands; Amsterdam UMC, Vrije Universiteit Amsterdam, Department of Human Genetics, De Boelelaan 1117, Amsterdam, The Netherlands; Amsterdam UMC, Vrije Universiteit Amsterdam, Department of Human Genetics, De Boelelaan 1117, Amsterdam, The Netherlands; Department of Medical Biology, Amsterdam UMC, University of Amsterdam, Amsterdam, The Netherlands; Amsterdam UMC, Vrije Universiteit Amsterdam, Department of Human Genetics and Cancer Center Amsterdam, De Boelelaan 1117, Amsterdam, The Netherlands; Amsterdam UMC, Vrije Universiteit Amsterdam, Department of Urology and Cancer Center Amsterdam, De Boelelaan 1117, Amsterdam, The Netherlands; Amsterdam UMC, Vrije Universiteit Amsterdam, Department of Human Genetics and Cancer Center Amsterdam, De Boelelaan 1117, Amsterdam, The Netherlands; Amsterdam UMC, Vrije Universiteit Amsterdam, Department of Human Genetics, De Boelelaan 1117, Amsterdam, The Netherlands

## Abstract

Birt–Hogg–Dubé syndrome (BHD) is an autosomal dominant disorder characterized by fibrofolliculomas, pulmonary cysts, pneumothoraces and renal cell carcinomas. Here, we reveal a novel hereditary disorder in a family with skin and mucosal lesions, extensive lipomatosis and renal cell carcinomas. The proband was initially diagnosed with BHD based on the presence of fibrofolliculomas, but no pathogenic germline variant was detected in *FLCN*, the gene associated with BHD. By whole exome sequencing we identified a heterozygous missense variant (p.(Cys677Tyr)) in a zinc-finger encoding domain of the *PRDM10* gene which co-segregated with the phenotype in the family. We show that *PRDM10^Cys677Tyr^* loses affinity for a regulatory binding motif in the *FLCN* promoter, abrogating cellular FLCN mRNA and protein levels. Overexpressing inducible *PRDM10^Cys677Tyr^* in renal epithelial cells altered the transcription of multiple genes, showing overlap but also differences with the effects of knocking out *FLCN*. We propose that *PRDM10* controls an extensive gene program and acts as a critical regulator of *FLCN* gene transcription in human cells. The germline variant *PRDM10^Cys677Tyr^* curtails cellular folliculin expression and underlies a distinguishable syndrome characterized by extensive lipomatosis, fibrofolliculomas and renal cell carcinomas.

## Introduction

Birt–Hogg–Dubé syndrome (BHD, MIM #135150) is an autosomal dominant inherited disorder characterized by fibrofolliculomas (FF), pulmonary cysts, pneumothoraces and renal cell carcinoma (RCC) (1–3). BHD is caused by loss-of-function pathogenic germline variants (PGV) in the *FLCN* gene, encoding the folliculin protein (4). As BHD-associated RCCs arise upon loss of the wild-type (WT) *FLCN* allele, usually due to a somatic second hit, folliculin functions as a tumor suppressor in the kidney (5). Epigenetic silencing of the WT *FLCN* allele in RCC has also been reported, but the factors critically controlling *FLCN* gene transcription are not known (6). Folliculin is amongst others involved in repressing TFEB/TFE3 transcription factor activity (7–10) and a non-canonical interferon response (11). Indeed, biallelic FLCN inactivation directs RCC formation in transgenic mice in a manner dependent on TFEB function (7). However, the exact roles of folliculin and the mechanisms by which reduced cellular folliculin levels cause the tissue-specific clinical features observed in BHD are not understood.

BHD can be diagnosed by a PGV in the *FLCN* gene, by the presence of at least five (histologically confirmed) FF, or by a combination of minor criteria including pulmonary cysts and RCC (12). BHD should be considered in patients with bilateral or multifocal RCC, chromophobe or hybrid oncocytic kidney tumors, occurrence of RCC before 50 years of age, bilateral basal pulmonary cysts and recurrent pneumothorax, especially when a combination of these has occurred in one family (13). A PGV in *FLCN* is found in the vast majority (84–91%) of patients with the clinical diagnosis of BHD (14–16). The remaining BHD patients might have an unidentifiable variant in *FLCN*, for example in a deep intronic region, or a PGV in a yet unknown gene.

Here, we present clinical and molecular data from a large family in which the proband was clinically diagnosed with BHD based on the presence of FF, but without a PGV in the *FLCN* gene. The phenotype was also distinct from BHD with more skin and mucosal lesions, extensive lipomatosis, and the absence of pulmonary manifestations. By whole exome sequencing (WES), we identified a heterozygous missense variant in *PRDM10* which co-segregated with the phenotype. We show that *PRDM10* acts as a critical transcriptional regulator of *FLCN* expression in human cells.

## Results

**Figure 1:**
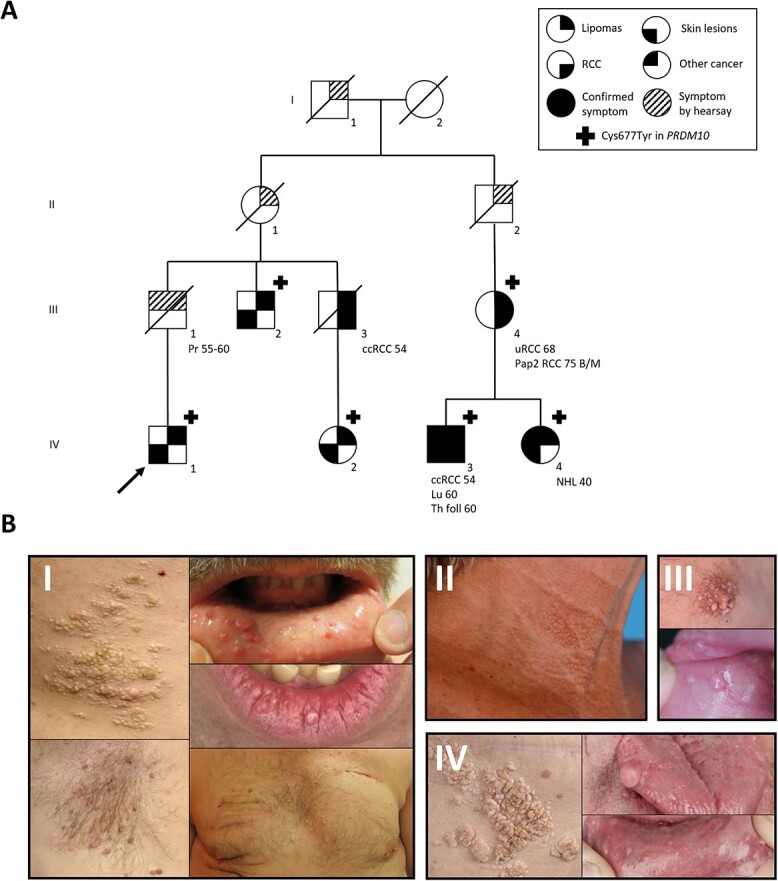
Pedigree and photos of skin and mucosal phenotype. (**A**) Pedigree of the family. Pr; prostate cancer, uRCC; unclassified renal cell carcinoma, B/M; bilateral and multifocal, ccRCC; clear cell renal cell carcinoma, Pap2 RCC; papillary type 2 renal cell carcinoma, Th folll; follicular thyroid carcinoma, Lu; lung carcinoma, NHL; non-Hodgkin lymphoma. The proband is indicated by an arrow. (**B**) Photos of affected patients. I (patient IV-1): partly confluent papules on the flank, skin tags in the armpit, intraoral papules, papules on the lip and multiple skin-colored papules on the trunk. II (patient IV-4): papules in the neck. III (patient IV-3): papules on the nipple, intraoral papules. IV (patient III-2): partly confluent papules on the flank and intraoral papules.

An overview of the extended pedigree of the family is shown in Figure 1A. Informed consent for publication of clinical data, photographs (if applicable) and use of tissue was obtained from patients III-2, III-4, IV-1, IV-2, IV-3 and IV-4.

### Clinical description

The proband (IV-1) was first evaluated at the age of 33 years because of skin and mucosal lesions and multiple lipomas of the trunk. The skin lesions consisted of multiple skin-colored papules in the face and on the trunk, skin tags on the trunk and an area on the right flank with multiple, small, partly confluent, yellow/white papules. Also, there were some intra-oral papules and small papules on the lips. Multiple biopsies were taken and one lesion showed the typical histologic features of FF. Other skin lesions were consistent with perifollicular fibromas. No pulmonary cysts were detected by a chest computed tomography (CT). Magnetic resonance imaging of the kidneys showed a small cyst in the left kidney. At re-evaluation at age 48 years, more than 50 lipomas had been surgically removed and many were still present. Most were located on the trunk and the proximal limbs. Histologic evaluation had repeatedly shown common lipomas without specific features. The proband reported severe pain limiting daily physical activity, possibly related to the many lipomas that had been removed or to those still present.

**Figure 2:**
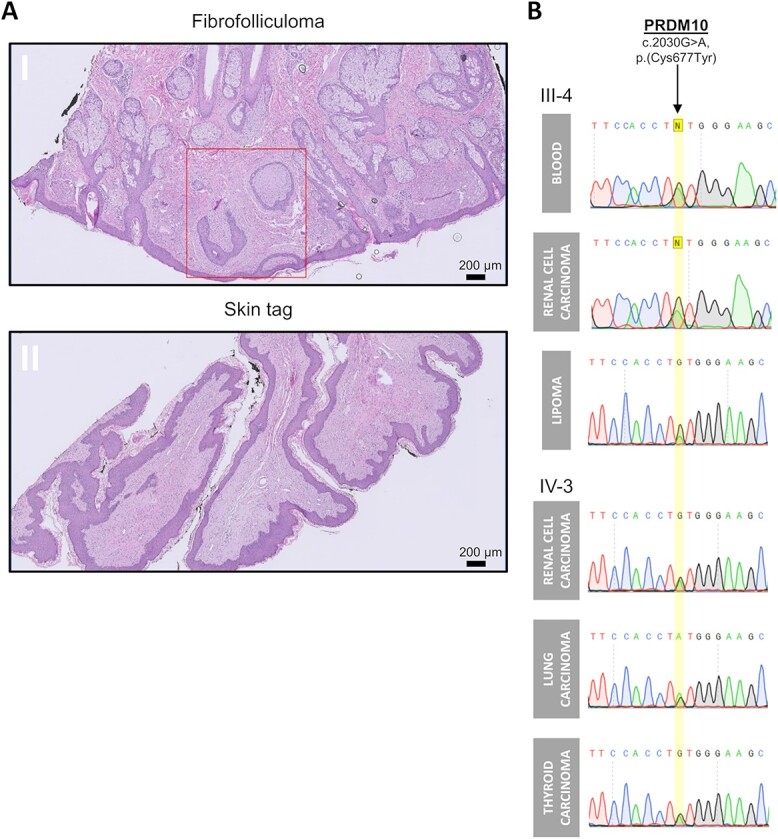
Histology of skin and mucosal phenotype and PRDM10 sequence analyses. (**A**) Histology of skin lesions from patient IV-3: panel I is H&E staining of skin biopsy of the cheek showing strands of epithelium surrounded by stromal cells in loose connective tissue with mucin. This histological picture is consistent with a diagnosis of a fibrofolliculoma (squared red). Panel II is H&E staining of scrotum biopsy showing a skin tag of fibrous stroma covered by squamous cell epithelium. Scale bar is 200 μm. (**B**) Sanger sequencing on DNA isolated from tissues derived from family members (III-4) and (IV-3) confirms the *PRDM10* c.2030G > A mutation without loss of the second *PRDM10* allele.

The proband reported a family history of lipomas, skin lesions and RCC in many family members, of which III-2, IV-2, IV-3 and IV-4 were also clinically evaluated. All of these family members had comparable skin lesions and lipomas. Images of the skin and mucosal phenotypes are shown in Figure 1B. Biopsies histologically confirmed the presence of FF in patients IV-3 and IV-4 (Fig. 2A). As in the proband, no pulmonary cysts were detected by a chest CT of patients III-4 and IV-3. Three affected family members were diagnosed with RCC: III-3 died of clear cell RCC at age 54 years, IV-3 had clear cell RCC at age 54 years and III-4 had bilateral and multifocal RCC of papillary and unclassified histology at age 68 years. In addition to the pedigree shown in Figure 1A, the proband mentioned nine more family members affected with lipomas. Of these, one had a history of prostate cancer and two had an unknown type of cancer at age 49 and 56 years, respectively. Another family member was mentioned to have a skin phenotype without lipomas. These additional family members did not consent with genetic testing or publication of clinical data. Several other types of cancer occurred in affected family members, namely lung adenocarcinoma, follicular thyroid cancer and non-Hodgkin’s lymphoma.

### Genetic testing

No PGV was detected in *FLCN* in the proband by conventional Sanger sequencing and multiplex ligation dependent probe amplification (MLPA). To investigate whether a shared haplotype of the *FLCN* gene co-segregated within the family, we employed microsatellite marker analysis using five microsatellite markers flanking *FLCN* (Supplementary Material, Fig. S1). A common haplotype could be identified in all affected individuals, except for individual IV-2. This individual has different haplotypes compared to all the other affected (including the D17S2196 marker closest to *FLCN* at 200kbp distance), suggesting that a common *FLCN* haplotype is unlikely to underlie the phenotype in this family.

Even though no other features of PTEN hamartoma tumor syndrome were present, Sanger sequencing and MLPA of *PTEN* were performed in IV-2 but no variants predicted to be pathogenic were detected. The proband and two other affected family members (III-2 and IV-2) consented for WES and the results were analyzed for shared rare variants. A list of all detected shared rare variants with their considerations is shown in Supplementary Material, Table S1. Four shared predicted missense variants were detected, of which two did not co-segregate with the phenotype and one had a relatively high frequency in GnomAD (17). Therefore, we considered the remaining missense variant in *PRDM10* (NM_020228.3; c.2030G > A, p.(Cys677Tyr)) to be the most likely causal variant. Importantly, this *PRDM10* variant was detected in a fourth affected family member (III-4) and subsequently also identified in her affected children IV-3 and IV-4. Thus *PRDM10^Cys677Tyr^* co-segregated with the phenotype within the family (nine informative meioses). When all non-genotyped individuals are set to an unknown genotype, this resulted in a Logarithm of Odds (LOD) score of 0.18, while the LOD score was 2.7 when all non-genotyped affected individuals were set to carry the *PRDM10* variant. This latter LOD score is approximately the maximal LOD score that can be obtained from a family with this structure.

Before family members IV-3 and IV-4 were linked to the proband, a WES-based hereditary cancer gene panel (Supplementary Material, Table S2) was performed in IV-3, but no other potential PGVs were detected. The identified *PRDM10* variant is present in GnomAD (v2.1.1, all exomes) with an allele frequency of 4.8e-4% (17). The Grantham score for cysteine and tyrosine is 194 and the variant is predicted to be deleterious by SIFT but unlikely to interfere with protein function by Align-GVGD (18–20). The cysteine at this location is highly conserved from mammals to zebrafish.

To assess *PRDM10* loss of heterozygosity, DNA was isolated from RCC and lipoma tissue from III-4 and from RCC, lung carcinoma and follicular thyroid carcinoma tissue from IV-3. Sanger sequencing displayed the *PRDM10* c.2030G > A, p.(Cys677Tyr) variant without loss of the second allele (Fig. 2B).

### PRDM10^Cys677Tyr^  *in vitro* model

**Figure 3:**
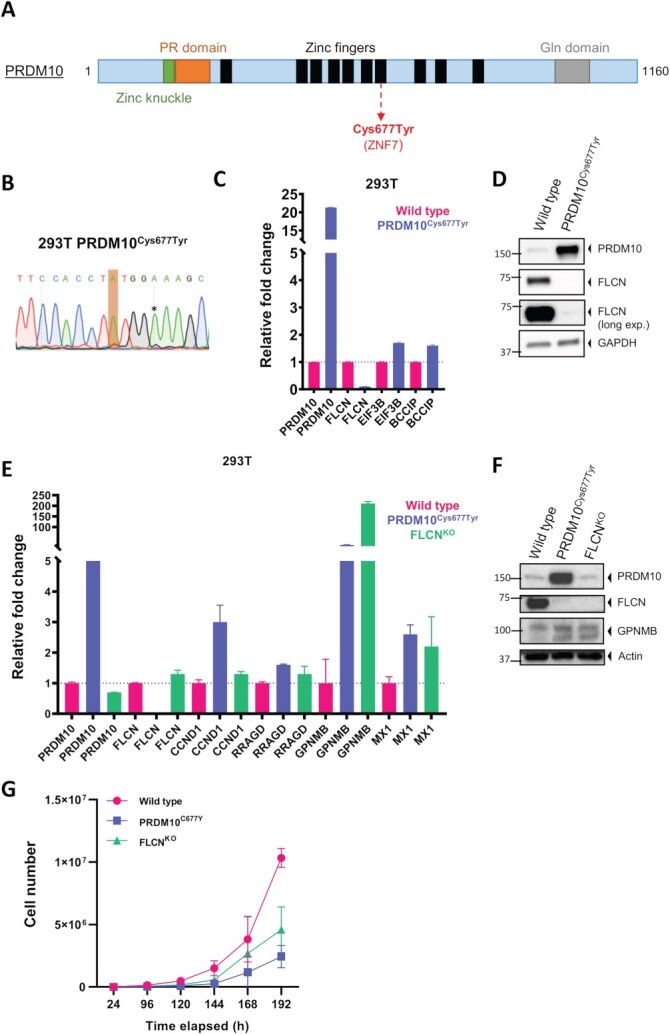
PRDM10^Cys677Tyr^ phenocopies cellular effects of FLCN loss in human embryonic kidney cells. (**A**) Schematic presentation of PRDM10 with known domains. Variant Cys677Tyr is located in seventh zinc finger and indicated in red. There are seven isoforms of PRDM10 described at Uniprot, the longest is 1160. (**B**) Creation of endogenous mutants in 293 T cells. Sanger sequence chromatogram of DNA derived from CRISPR prime edited cells shows a homozygous endogenous PRDM10^Cys677Tyr^ c.2030G > A mutation (indicated in orange). To prevent further genome editing, a silent mutation (indicated by asterisk) was introduced to disrupt the PAM site. (C) qPCR (*n* = 4) shows downregulation of FLCN expression upon PRDM10^Cys677Tyr^. Expression levels of EIF3B and BCCIP are increased. (**D**) Western blot (*n* = 3) shows downregulation of FLCN protein upon PRDM10^Cys677Tyr^. We detected higher PRDM10 protein levels in the PRDM10^Cys677Tyr^ mutant as compared to the wild type (WT). GAPDH was used as a loading control. (**E**) qPCR showed that PRDM10^Cys677Tyr^ resulted in upregulation of specific genes, which are also induced upon FLCN loss (FLCN^KO^) in 293 T cells. Results are representative for three independent experiments with at least two technical replicates. To determine quantitative gene expression levels, data were normalized to the geometric mean of two housekeeping genes. Note that FLCN mRNA is still detectable in the FLCN^KO^ cell line, possibly due to incomplete nonsense-mediated mRNA decay or a transcriptional feedback mechanism. (**F**) Western blots (*n* = 2) of 293 T mutant cell lines. FLCN protein is absent in both PRDM10^Cys677Tyr^ and FLCN^KO^ 293 T. Induction of GPNMB in PRDM10^Cys677Tyr^ was similar to induction levels observed in FLCN^KO^ cells. Actin was used as loading control. (**G**) Both PRDM10^Cys677Tyr^ and FLCN^KO^ cells grew slower when compared to wild type. Cells were seeded in equal densities and total cell number was counted for six consecutive days (*n* = 2).

The *PRDM10^Cys677Tyr^* variant is located in the seventh C2H2 zinc finger domain of the PRDM10 protein (Fig. 3A). We used Clustered Regularly Interspaced Short Palindromic Repeats (CRISPR)-based prime editing (PE) to establish a homozygous *PRDM10^Cys677Tyr^* human embryonic kidney cell line 293 T (HEK293T) to model this variant without interference of the WT allele (Fig. 3B). First, we assessed cellular localization of both WT and mutant PRDM10 using immunofluorescence. As shown in Supplementary Material, Figure S2A, PRDM10 was predominantly localized in the nucleus in both conditions, in line with its proposed transcriptional regulatory function (21). Using quantitative reverse transcriptase-PCR (q RT-PCR) and western blotting (Fig. 3C and D), we found higher expression of mutant PRDM10 when compared to WT PRDM10 at both the mRNA and protein level. *Flcn*, together with *Eif3b* and *Bccip*, has been reported as a putative transcriptional target gene bound and regulated by *Prdm10* in mice (21). Analysis of mRNA expression levels of *FLCN*, *EIF3B* and *BCCIP* in our cell model revealed a significant decrease in *FLCN* expression in the *PRDM10^Cys677Tyr^* cells, while the expression levels of *EIF3B* and *BCCIP* were increased (Fig. 3C). Next, we assessed whether *PRDM10^Cys677Tyr^* affected folliculin protein levels by western blot (Fig. 3D). In line with the strong decrease of *FLCN* mRNA expression levels, folliculin protein was almost undetectable in *PRDM10^Cys677Tyr^*. For validation, the effects on *FLCN* expression were confirmed in a second, independent *PRDM10^Cys677Tyr^* 293 T cell line clone (Supplementary Material, Fig. S2B and C). Moreover, we did not observe significant changes in *PTEN* expression in the *PRDM10^Cys677Tyr^* cell lines (Supplementary Material, Fig. S2C).

### Comparison to folliculin knockout

Recently, we showed that folliculin loss in renal epithelial cells (RPTEC/TERT1) upregulates transcription of many genes, including *RRAGD*, *GPNMB*, *CCND1* and *MX1* (11). In *PRDM10^Cys677Tyr^* 293 T cells, we observed qualitatively similar effects on the induction of these genes on mRNA levels as well as induction of GPNMB protein when compared to folliculin knockout 293 T cells (Fig. 3E and F). Note that *FLCN* transcript is still detectable in the *FLCN^KO^* cell line (Fig. 3E) yet folliculin protein expression is disrupted by a premature stop codon resulting from the gene editing procedure (Fig. 3F). This was also observed previously (11). Detection of FLCN mRNA may relate to incomplete nonsense-mediated mRNA decay combined with active *FLCN* gene transcription. Growth curves of *PRDM10^WT^*, *PRDM10^Cys677Tyr^* and *FLCN^KO^* 293 T show that both mutant cell lines display a similar alteration in growth properties when compared to WT cells (Fig. 3G).

### PRDM10^Cys677Tyr^ ChIP-qPCR

**Figure 4:**
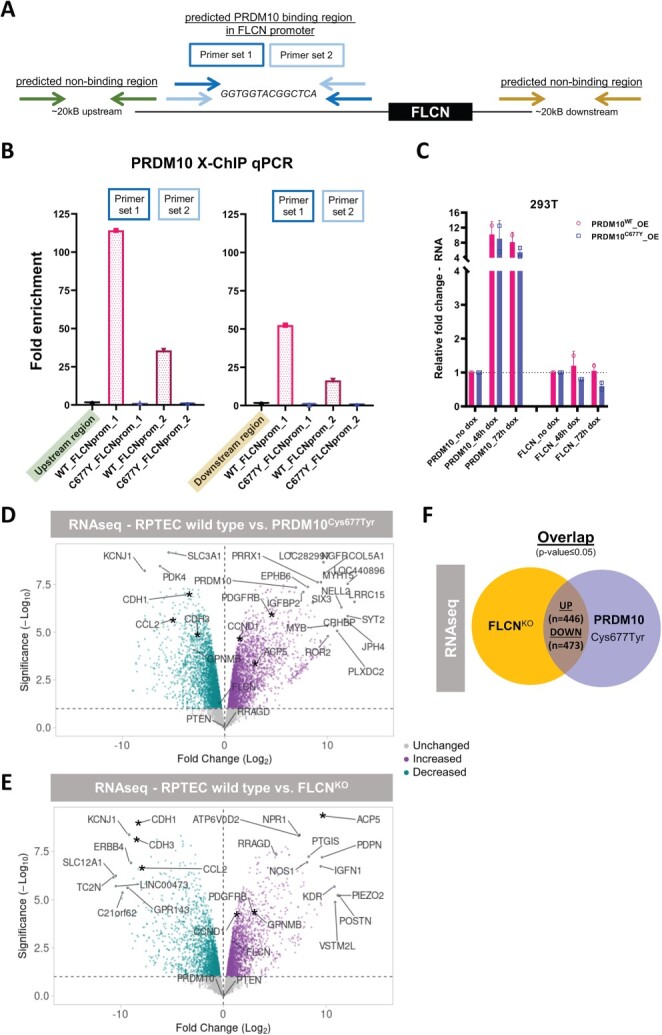
FLCN is a transcriptional target of PRDM10. (**A**) Schematic overview of primer locations used for X-ChIP qPCR experiments as described in (B). (**B**) FLCN promoter binding by PRDM10 was assessed by PRDM10 X-ChIP of 293 T PRDM10 wildtype and Cys677Tyr (C677Y) mutant cells. Fold enrichment of binding capacities between wild-type and C677Y mutant PRDM10 was determined by qPCR, showing that the mutation strongly diminished promoter binding. Bar graphs are representative of differences observed in three independent ChIP experiments, with two technical replicates per qPCR, and normalized to input and a predicted non-binding region ~20 kB upstream (left) or downstream (right) of the FLCN promoter. Two different primer sets surrounding the predicted PRDM10 motif (GGTGGTACGGCTCA) in the FLCN promoter were used. (**C**) qPCR (*n* = 2) shows that inducible overexpression (OE) of PRDM10^Cys677Tyr^ slightly repressed FLCN expression within 72 h, while this effect did not occur upon overexpression of PRDM10^WT^ in 293 T cells. (**D**) Volcano plot showing gene expression changes upon five days induction of PRDM10^Cys677Tyr^ in RPTEC/TERT1. 6087 genes are differentially expressed (*P*-value ≤ 0.05). Top 20 of most significant differential genes are indicated, plus, as a reference, these marker genes: PRDM10, FLCN, GPNMB, RRAGD, PTEN, CCND1, CDH1, CDH3, ACP5, PDGFRB and CCL2. Asterisks indicate genes of potential interest overlapping between FLCN^KO^ and PRDM10^Cys677Tyr^ cell lines. (**E**) Volcano plot showing gene expression changes upon FLCN^KO^ in RPTEC/TERT1. A total of 3703 genes are differentially expressed (*P*-value ≤ 0.05). Top 20 of most significant differential genes are indicated, plus, as a reference, these marker genes: PRDM10, FLCN, GPNMB, RRAGD, PTEN, CCND1, CDH1, CDH3, ACP5, PDGFRB and CCL2. Asterisks indicate genes of potential interest overlapping between FLCN^KO^ and PRDM10^Cys677Tyr^ cell lines. (**F**) Overlap of gene expression changes upon FLCN loss or PRDM10^Cys677Tyr^ induction in RPTECs. A total of 446 genes are significantly upregulated in both conditions and 473 genes are significantly downregulated in both conditions. Gene lists are provided as Supplementary Material, Table S3.

We hypothesized that *FLCN* is a direct transcriptional target of *PRDM10* in human cells. To test whether the Cys677Tyr variant alters PRDM10 binding to the *FLCN* promoter region, we performed ChIP-qPCR experiments. For these experiments, two independent primer sets surrounding the predicted PRDM10 binding motif (GGTGGTACGGCTCA) (21) were designed (Fig. 4A). Enrichments were calculated compared to a random region ~20 kB upstream (Fig. 4B, left plot) or downstream (Fig. 4B, right plot) of the *FLCN* promoter region. Fold enrichments of *FLCN* promoter DNA bound by PRDM10 in WT (*PRDM10^WT^*) or *PRDM10^Cys677Tyr^* mutant cells show that the *FLCN* promoter region is bound by PRDM10^WT^. Clearly, *PRDM10^Cys677Tyr^* results in a strong decrease in *FLCN* promoter binding, hampering its transcription.

### PRDM10^Cys677Tyr^ inducible overexpression

To investigate possible dominance of *PRDM10^Cys677Tyr^* over *PRDM10^WT^*, we performed experiments with inducible overexpression (OE) of *PRDM10^Cys677Tyr^* in the presence of endogenous, WT PRDM10 in 293 T cells. By qPCR, it was shown that OE of *PRDM10^Cys677Tyr^* slightly represses *FLCN* expression within 72 h, and this effect does not occur upon OE of *PRDM10^WT^* (Fig. 4C). However, a specific decrease of folliculin protein was not yet visible after 72 h of *PRDM10^Cys677Tyr^* OE (Supplementary Material, Fig. S2D and E). Prolonged, 120-h induction of *PRDM10^Cys677Tyr^* in RPTECs modestly repressed *FLCN* but strongly altered the expression of multiple other genes (6087 differentially expressed genes, Fig. 4D). The gene expression changes induced by PRDM10^Cys677Tyr^ showed similarities, but also differences, with the effects of knocking out *FLCN* (3703 differentially expressed genes, Fig. 4E). These results show that *PRDM10^Cys677Tyr^*, apart from curtailing *FLCN* expression, alters the expression of a variety of other genes, too. Furthermore, the transcriptional effects of increased *PRDM10^Cys677Tyr^* levels show overlap but are not identical to the gene expression patterns resulting from loss of *FLCN* (Fig. 4F and Supplementary Material, Table S3).

## Discussion

We here present a family initially clinically diagnosed with BHD but without a detectable PGV in *FLCN*. Many years later, the family was re-evaluated upon their own request and additional genetic testing was performed. A missense germline variant in the *PRDM10* gene was detected that co-segregated with the phenotype within the family. The presence of nine informative meiosis equals a probability of 1/512 that co-segregation of the variant in this family occurred by chance (22). Therefore, we considered the *PRDM10* variant as the most likely causal variant underlying this novel syndrome. This is the first association of a germline variant in *PRDM10* with a Mendelian human disease (MIM #618319).

The clinical features of the here presented family overlap with BHD, and a comparison of the clinical phenotype of the here described family and BHD is shown in Table 1. The initial clinical diagnosis of BHD was based on the presence of FF and multiple perifollicular fibromas, which are lesions in the spectrum of FF (23,24). However, the location, color and morphology of the skin lesions were partly different from those in BHD. Since the initial presentation, three family members had developed RCC, which is one of the hallmark manifestations of BHD. Phenotypically, there were two main differences with BHD. First, no pulmonary cysts were present in three patients who underwent a chest CT, whereas pulmonary cysts are present in 70–100% of BHD patients (2,16,25–30). Second, extensive lipomatosis was present in many patients in the family described here, which is not an established feature of BHD. In our cohort of more than 300 BHD and BHD-like patients, we have no other families with such extensive lipomatosis and no families with definite BHD without an identifiable *FLCN* pathogenic variant. Our cohort does contain some families with BHD-like features in which we have attempted to identify the genetic cause, for example by WES, but in none a (possible) pathogentic variant in *PRDM10* was identified*.* Multiple lipomas can occur in the presumed autosomal dominant conditions familial multiple lipomatosis (MIM #151900) and Dercum disease (MIM #103200) (31). No genes associated with these diseases have been identified yet. Multiple lipomas can also occur in PTEN hamartoma tumor syndrome (MIM #158350), which is an autosomal dominant condition caused by PGVs in the *PTEN* gene (32,33). We considered the diagnosis of PTEN hamartoma tumor syndrome unlikely, since no other features of PTEN hamartoma tumor syndrome were present and no PGV was detected in *PTEN.*

**Table 1:** Comparison between the phenotype of Birt–Hogg–Dubé syndrome and the family presented here

	Birt–Hogg–Dubé syndrome	*PRDM10* family
Renal cell carcinoma		
Prevalence	7–34% (2,15,16,25–27,34)	3/10 carriers and obligate carriers 3/22 including family members affected with lipomas/skin phenotype by hearsay
Mean age at first RCC	50 (16,26,34,35)	59
Subtype	Most subtypes can occur Subtypes with chromophobe component most common (2,15,26,34,36,37)	Clear cell, papillary type 2, unclassified
Pneumothorax Prevalence	22–74% (2,14–16,25,27–30)	0
Pulmonary cysts Prevalence	70–100% (2,16,25–30)	0/3
Skin phenotype		
Prevalence	80–90% (in Europe and USA) (15,27,28)	All assessed patients (*n* = 5)
Morphology	White to yellow papules on the face, neck and upper trunk (1,38)	White to yellow and skin-colored papules on the face, neck, trunk, nipples and scrotum. Areas of partly confluent papules on the trunk Mucosal papules
Histology	Fibrofolliculoma spectrum (38–41)	Fibrofolliculoma spectrum
Lipomas	No proven association	All assessed patients (*n* = 5) 9 more family members by hearsay

We also performed WES analysis in two other familial cases of suspected BHD without an identifiable PGV in *FLCN*, but no other variants in *PRDM10* were detected.

*PRDM10* is 1 of 19 *PRDM* genes currently known in humans (42). It belongs to the PR/SET (positive regulatory domain-binding factor 1 (**P**RDI-BF1) and retinoblastoma interacting zinc finger (**R**IZ) homology domain containing/**S**u(var)3-9, **e**nhancer-of-zeste and **t**rithorax) transcription factor family which share a conserved N-terminal PR domain that has similarities with the lysine methyltransferase SET domain, followed by variable C2H2-type zinc finger repeats (43,44). In mice, homozygous knockout of *Prdm10* leads to preweaning lethality and *Prdm10* functions as a sequence-specific transcription factor which is essential in supporting global translation during early development (21). In humans, PRDM10 is expressed in almost all tissues including skin, kidney and adipose tissue (45,46).

We found a significant decrease of *FLCN* expression in the *PRDM10^Cys677Tyr^* cells. Since *Flcn* was suggested to be a target of *Prdm10* in mice (21) and because the Cys677Tyr variant is located in the DNA-binding domain of *PRDM10*, we hypothesized that *FLCN* could be a direct transcriptional target of PRDM10 in humans. Indeed, we observed that WT PRDM10 binds to the *FLCN* promoter region and that *PRDM10^Cys677Tyr^* results in a strong decrease in promoter binding, curtailing FLCN expression in human cells. These results identify PRDM10 as an upstream regulator of the folliculin tumor suppressor. Indeed, *PRDM10^Cys677Ty^* resulted in several effects comparable to those of the loss of folliculin *in vitro*, such as induction of GPNMB protein and reduced proliferation. While reduced proliferation may seem paradoxical given the proven role of *FLCN* as a tumor suppressor, this observation is in line with our previous studies of *FLCN^KO^* RPTECs, which experience a growth disadvantage upon folliculin loss due to induction of a non-canonical interferon response, counterbalancing the TFE3/TFEB-directed hyperproliferation upon folliculin loss (11).

So, in the family described here, a reduction in folliculin expression levels caused by the *PRDM10* variant might largely explain the occurrence of FF and RCC, phenocopying BHD. However, the absence of a pulmonary phenotype in this family may reflect a specific role of *PRDM10*, where tissue specific expression patterns may play a role. Indeed, the protein levels of PRDM10 in human lung may be lower than in tissues that are affected in this family (46,47). Hypothetically, *FLCN* expression in the lungs may be under control of additional transcription factors, overruling the effects of the *PRDM10* variant. Although the development of the pulmonary phenotype in BHD has not been investigated in great detail, it was shown that *FLCN* deficiency hampers the cellular E-cadherin-LKB1-AMPK axis and that folliculin is required for alveolar epithelial cell survival (48). It will be interesting to investigate whether PRDM10 could act as an upstream regulator of the FLCN-E-cadherin-LKB1-AMPK axis in lung cells and how the variant may affect this pathway.

The lipomatosis could be either a direct effect of the *PRDM10* variant, for example by gene expression changes other than *FLCN* reduction, or it may be an adipose tissue-specific effect of folliculin repression and downstream TFE3/TFEB activation, which is described to play a role in adipose tissue (49–52). Heterozygous loss of *Prdm10* expression was reported to result in a reduction of fat mass in male mice (53), confirming role for *Prdm10* in adipose tissue. Based on these mouse data, with an apparent opposite adipose tissue phenotype than observed in the family reported here, it could be hypothesized that the lipomatosis in this family is not the result of reduced folliculin expression, but due to a change in the expression of a specific gene target of *PRDM10^Cys677Tyr^* (Figs 3C and 4D).

In addition to RCC, several other types of cancer have occurred in the affected members of this family. Future research will be needed to understand whether these might be caused by the *PRDM10* variant. Alterations in the *PRDM* genes are linked to various tumors, as recently reviewed by Casamassimi *et al.*, so it is possible that the variant in this family may predispose for cancers other than RCC as well (54). Also, there is some evidence for a role of somatic aberrations of PRDM10 in cancer. *PRDM10* gene fusions with either *MED12* or *CITED2* have been reported in a small proportion of undifferentiated pleomorphic sarcomas. These tumors have a relatively benign course, a distinct expression pattern and no or limited other mutations or numerical chromosomal aberrations (55,56). *PRDM10* increases B-cell lymphoma-2 (Bcl-2) expression *in vitro* (57). The Bcl-2 gene is upregulated in a wide variety of cancers including RCC and selective Bcl-2 inhibition was reported as a potential strategy in the treatment of RCC (58,59). Furthermore, *PRDM10* is overexpressed in breast, colon and liver cancer samples (42,60). A relative OE in kidney cancer samples was also present, but the difference with normal tissue was not significant (42). These data also suggest that PRDM10 may play a role in cancer, but the mechanism requires further studies.

Remarkably, in our *in vitro* experiments in human cells, is that the mutant PRDM10 was expressed at higher levels than the WT PRDM10. We have not studied the mechanism underlying the increased expression in detail, but a possible explanation for the increased PRDM10 protein levels is the existence of a feedback loop leading to increased *PRDM10* promoter activity, as indicated by the increased mutant *PRDM10* mRNA levels. In the Eukaryotic Promoter Database (61,62), we noted that the predicted *PRDM10* binding motif is also present in its own promoter region, suggesting that *PRDM10* may be capable of regulating its own expression, and that this may be affected by the Cys677Tyr variant. Alternatively, another upstream regulator of *PRDM10* expression could be affected by the Cys677Tyr variant which results in an increase of *PRDM10* expression, or the Cys677Tyr variant increases *PRDM10* mRNA stability.

Whether *PRDM10* functions as a more canonical tumor suppressor gene, requiring functional inactivation of the second allele for development of neoplasms, is not clear yet. In the family described here, the tumors and lipoma lesions investigated did not show loss of the second *PRDM10* allele, although epigenetic silencing or a second hit of the second *PRDM10* allele was not excluded. *PRDM10^Cys677Tyr^* also affects gene expression in a way independent of the reduced folliculin levels. Based on these combined results, we propose that PRDM10^Cys677Tyr^ could act as either a neomorphic or hypomorphic allele, and may have a dominant effect over the second *PRDM10* allele, at least for some transcriptional targets. It is likely that external factors or (epi)genetic aberrations in other genomic regions played a role in the development of the observed neoplasms, too.

In conclusion, we identified a distinguishable syndrome partly overlapping with BHD, consisting of multiple lipomas, FF and RCC, caused by a missense variant in *PRDM10*. To further pinpoint the roles of PRDM10 in different tissues and its role as a hereditary cancer gene, future studies in custom-made *in vivo* models are required. Our observations can serve as the basis for further functional studies into the roles of *PRDM10* as a disease gene, and provide further insight into BHD, lipomatosis and RCC pathogenesis.

## Materials and Methods

### Germline genetic testing

All germline genetic testing (Sanger sequencing, MLPA and WES) was performed in the diagnostic setting in laboratories in the Netherlands accredited in accordance with ISO15189. Segregation analysis of the *PRDM10* variant in III-9, IV-14 and IV-15 was performed by Sanger sequencing using the following primers: Fw 5’ CCCCGATAAACTGCGACT 3′ and Rev 5’ GAGAACCACCTTGGGCTG 3′. WES was performed as described before (63). Median and average coverage of the exome target region was >100x for each sample. Variant prioritization was performed using Alissa Interpret (Agilent Technologies, Santa Clara, CA, USA). In short, a classification tree was used to select for variants present in all three affected individuals and virtually absent in control cohorts dbSNP build 142 (http://www.ncbi.nlm.nih.gov/projects/SNP), 1000 Genomes Phase 3 release v5.20130502, and ESP6500 (http://evs.gs.washington.edu/EVS/) as well as in house controls. Prerequisite was that these variants had been genotyped in at least 200 alleles. Subsequently, the remaining variants were manually inspected and further prioritized based on literature, predicted (deleterious) effects on protein function by e.g. truncating the protein, affecting splicing, amino acid change and evolutionary conservation.

### LOD score calculation

A two-point LOD score calculation was performed using Superlink-Online SNP (64) for the *PRDM10* variant using the pedigree and phenotype as depicted in Figure 1A.

### Microsatellite marker haplotypes analysis

Microsatellite marker haplotypes analysis was performed using five microsatellite markers flanking the *FLCN* gene on an ABI 3700 Genetic Analyzer (Applied Biosystems, Carlsbad, CA, USA) (65). Data were analyzed using the genemapper 5.0 software (Applied Biosystems, Carlsbad, CA, USA).

### Study approval

The Medical Research Involving Human Subjects Act (WMO) did not apply to this study, since all genetic testing was performed in the diagnostic setting. Informed consent for publication of clinical data, photographs and use of tumor tissues was obtained.

### DNA isolation and sanger sequencing

DNA was extracted from blood and Formalin-Fixed Paraffin-Embedded (FFPE) tissues and equal amounts of DNA were amplified by PCR. Tubes were placed in a thermal cycler (Veriti, Thermo Fisher Scientific Inc, Waltham, MA, USA) for amplification with specific PCR primer mixes (10 μm). PCR program used for amplification was 1 cycle of 94°C for 3 minutes, 5 cycles of 94°C for 30 sec, 65°C for 30 sec, 72°C for 120 sec, 30 cycles of 94°C for 30 sec, 60°C for 30 sec, 72°C for 2 minutes, 72°C for 10 minutes and ending in a rapid thermal ramp to 10°C. After PCR purification (QIAquick PCR Purification Kit, Qiagen, Germany) took place, samples were further analyzed by sequencing. Sequencing was either performed in-house or at Eurofins Genomics. For PCR and sequencing of *PRDM10*, following primers were used: Fw 5’ CCCGATAAACTGCGACTCCACAT 3′ and Rev 5’ GGTCCAGTTCATCAGAGGTGGGTG 3’.

### Cell culture and genome editing

Human embryonic kidney cells (HEK293T, ATCC CRL-3216™) were maintained in Dulbecco’s Modified Eagle Medium (DMEM, Gibco, Life Technologies, Thermo Fisher Scientific Inc, Waltham, MA, USA) supplied with 8% fetal bovine serum (FBS, F0804, Sigma-Aldrich, St. Louis, MO, USA). Renal proximal tubular epithelial cells (RPTEC/TERT1, ATCC CRL-4031™) were maintained in DMEM/F12 (Gibco, Life Technologies, Thermo Fisher Scientific Inc, Waltham, MA, USA) according to the manufacturer’s protocol with addition of 2% fetal bovine serum. Cell lines were cultured in a humidified atmosphere at 37°C and 5% CO_2_ and were regularly tested to exclude *Mycoplasma* infections.

To introduce the *PRDM10* variant endogenously, CRISPR PE was used (66). First, a doxycycline inducible PE protein plasmid, containing a Cas9 nickase fused to a RT domain, was cloned and stably expressed in 293 T cells. Then, cells were seeded in the presence of doxycycline (10 ng/ml, Sigma-Aldrich, St. Louis, MO, USA) and the next day pegRNA plasmid, designed to both introduce the PRDM10 c.2030G > A variant and disrupt the PAM-site (available on request), was transfected using Lipofectamine 3000 reagent (Thermo Fisher Scientific Inc, Waltham, MA, USA) into Cas9-RT expressing 293 T cells. The *FLCN* knockout cell lines were created using Synthego’s Synthetic cr:tracrRNA Kit and corresponding manual. Cas9/gRNA (FLCN_exon 4 GAGAGCCACGAUGGCAUUCA + modified EZ scaffold) RNP complexes were transfected transiently using Neon Electroporation System (Thermo Fisher Scientific Inc, Waltham, MA, USA). For all CRISPR experiments, cells were grown in limiting dilution in 96-wells plates to generate single cell clones and genome editing status was assessed by Sanger sequencing. Sequenced samples were analyzed by manual alignment and using the Synthego ICE analysis (ice.synthego.com) tool which gives a quantitative score of editing efficiency.

### Virus production and infection

To create inducible cell lines that overexpress PRDM10^WT^ (293 T) and PRDM10^Cys677Tyr^ (293 T and RPTEC/TERT1), lentiviral production and transduction took place according to the Lenti-X Tet-On 3G Inducible Expression System (Clontech, Takara Bio, Japan) technical manual. In short, PRDM10^WT^ and PRDM10^Cys677Tyr^ cDNA were cloned into the pLVX-Tre3G plasmid where after Tre3G-Cas9 and Tet3G lentiviral particles were produced in 293 T cells. For transduction, cells were seeded in a 6-wells plate. The next day growth media was changed for 1 ml media containing viruses. Cells were incubated overnight and after 24 hours media was replaced with 2 ml fresh media. The next day cells were transferred to 10 cm plates and Puromycin (3 μg/ml, Sigma-Aldrich, St. Louis, MO, USA) was added to select for successfully transduced cells.

### RNA isolation and quantitative RT-PCR

The High Pure RNA Isolation Kit (Roche, Penzberg, Germany) was used to extract RNA from the dry cell pellet. For qRT-PCR we used Biorad iScript cDNA Synthesis Kit and LightCycler 480 FastStart DNA Master SYBR Green I (Roche, Penzberg, Germany). Measurements were performed with LightCycler 480 System and corresponding software (Roche, Penzberg, Germany). To determine the quantitative gene expression data levels were normalized to the geometric mean of two housekeeping genes. All experiments were at least performed in duplicate with three technical replicates per experiment. Primer sequences used in this study are as follows:

**PRDM10** Fw 5’ CAGGAACTGAAGGTGTGGTATG 3’ Rev 5’ GCTCTCGAAGAACTTTCCTTTCT 3’

**CCND1** Fw 5’ GCGGAGGAGAACAAACAGAT 3’ Rev 5’ GAGGGCGGATTGGAAATGA 3’

**EIF3B** Fw 5’ GGAGACCGCACTTCCATATTC 3’ Rev 5’ CTTAGGAGACCAACGCACATAC 3’

**BCCIP** Fw 5’ AGAACCATATTGGGAGTGTGATTA 3’ Rev 5’ ACACTGGGTACCCTTTCTTTC 3’

**FLCN** Fw 5 ‘GGAGAAGCTCGCTGATTTAGAAGAGGA 3‘ Rev 5’ ACCCAGGACCTGCCTCATG 3′

**MX1** Fw 5’ GACAATCAGCCTGGTGGTGGTC 3’ Rev 5’ GTAACCCTTCTTCAGGTGGAACACG 3’

**GPNMB** Fw 5’ CCTCGTGGGCTCAAATATAAC 3’ Rev 5’ TTTCTGCAGTTCTTCTCATAGAC 3’

**RRAGD** Fw 5’ CCTGGCTCTCGTTTGCTTTGTCAG 3’ Rev 5’ GGGGTGGCTCTCTTTTTCTTCTGC 3’

**HPRT1** Fw 5’ TGACACTGGGAAAACAATGCA 3‘Rev 5 ‘GGTCCTTTTCACCAGCAAGCT 3‘

**TBP** Fw 5 ‘TGCACAGGAGCCAAGAGTGAA 3‘ Rev 5’ CACATCACAGCTCCCCACCA 3‘

### Immunoblot

For western blotting, dry cell pellets were lysed in RIPA lysis buffer (89900, Thermo Scientific) supplemented with protease and phosphatase inhibitors (Roche, Penzberg, Germany). Lysates were boiled at 70°C for 5 min in 1x NuPAGE LDS sample buffer (Novex NP0007, Thermo Fisher Scientific Inc, Waltham, MA, USA) with 10% 1 M DTT (Sigma) and equal amounts were separated by 4–15% sodium dodecyl sulfate–polyacrylamide gel electrophoresis (SDS-PAGE) (BioRad, Hercules, CA, USA) and blotted onto polyvinylidene fluoride transfer membranes (Merck Millipore, MA, USA). Subsequently, membranes were blocked for 1 hour at room temperature with 5% milk (ELK, Campina, Amersfoort, Netherlands) in TBST. The primary antibody incubation was overnight at 4°C in 2.5% milk in TBST. The following day, membranes were washed and incubated with appropriate secondary antibodies (Dako, Agilent, CA, USA) for 3 h at 4°C in 2.5% milk in TBST. After incubation, the membranes were thoroughly washed and bands were visualized by chemiluminescence (ECL prime, Amersham, VWR, Radnor, PA, USA) in combination with ChemiDoc Imaging Systems (BioRad Laboratories, CA, USA). The following antibodies were used according to individual datasheets: PRDM10 Bethyl (A303-204A, Bethyl laboratories, TX, USA), FLCN (D14G9, CST 3697S, Cell Signaling Technologies, MA, USA), GPNMB (AF2550-SP, R&D systems, MN, USA), PTEN (sc-7974, Santa Cruz, TX, USA), β-Actin (sc-47778, Santa Cruz, TX, USA) and GAPDH (sc-47724, Santa Cruz, TX, USA).

### ChIP-qPCR

For chromatin immunoprecipitation experiments, cells were cross-linked with 1% formaldehyde for 10 minutes at room temperature, quenched with 125 mm glycine and washed twice in cold PBS.

Chromatin extracts were obtained by consecutive rounds of lysis in LB1 (50 mm Tris, pH 7.5, 140 mm NaCl, 1 mm EDTA, 10% glycerol, 0.5% NP-40, 0.25% Triton X-100), LB2 (10 mm Tris, pH = 8.0, 200 mm NaCl, 1 mm EDTA, 0.5 mm EGTA) and LB3 (10 mm Tris, pH = 8.0, 100 mm NaCl, 1 mm EDTA, 0.5 mm EGTA, 0.1% Na-Deoxycholate, 0.5% N-lauroylsarcosine), supplemented with protease inhibitor cocktail (Roche, Penzberg, Germany). Chromatin DNA was sheared to a size range of 100–500 bp using sonication for 12,5 minutes (30 sec on, 30 sec off at high amplitude) using a Bioruptor sonicator (Diagenode, NJ, USA). Triton X-100 was added to a final concentration of 1% and lysates were cleared by centrifugation, where after 50 μl input sample was taken. Subsequently, 40 μl A/G agarose beads (sc-2003, Santa Cruz, TX, USA) and 5 μg PRDM10 antibody (pre-coupled overnight at 4°C) were added to sonicated chromatin and incubated overnight with rotation at 4°C. The next day, beads were washed with RIPA lysis buffer (89 900, Thermo Fisher Scientific Inc, Waltham, MA, USA) 10 times. To elute and reverse cross-links, samples were incubated with 200 μl elution buffer (2% SDS in TE buffer) overnight at 65°C then treated with proteinase K. DNA was then purified by phenol:chloroform extraction. ChIP and input DNA were measured using quantitative RT-PCR, performed as described above. Fold enrichments of binding capacities were calculated between WT and mutant PRDM10, and normalized to input and a predicted non-binding region ~20 kB upstream or downstream of the *FLCN* promoter. Two different primer sets surrounding the predicted PRDM10 motif (GGTGGTACGGCTCA) in the *FLCN* promoter were used:

 FLCN_promoter_set1_Fw 5’ CTGTGTTCCTGGGCTTGC 3’

 FLCN_promoter_set1_Rev 5’ CCGGGTTCAGGCTCTCA 3’

 FLCN_promoter_set2_Fw 5’ AGTTGTAGGACTCGGACTGTG 3’

 FLCN_promoter_set2_Rev 5’ AGCTGGCAGAACCAGGA 3’

 FLCN_promoter_upstream_Fw 5’ CAGTCTGGGCAACTAAGTAAGA 3’

 FLCN_promoter_upstream_Rev 5’ CAAGGGAACCTCCTGTTTCA 3’

 FLCN_promoter_downstream_Fw 5’ AGGTGTCAATGTCATGGAAGTTA 3’

 FLCN_promoter_downstream_Rev 5’ AGGAGATACTACAGGACCCATC 3’

### Immunofluorescent staining

293 T cells were grown on cover slips, fixed in 2% paraformaldehyde for 15 minutes at room temperature and subsequently in 70% ice cold EtOH for 1 hour. Next, cells were permeabilized in 0.3% Triton X-100 for 5 min, blocked in 3% BSA with 0.3% Triton X-100 for 45 minutes, incubated with rabbit anti-PRDM10 antibody (1:100, HPA026997, Atlas antibodies, Sweden) for 1.5 hour and secondary antibody for 1 hour at room temperature. Cells were mounted using ProLong™ Gold Antifade Mountant with DAPI (Invitrogen, Thermo Fisher Scientific Inc, Waltham, MA, USA) and examined using fluorescence microscopy (Leica, Germany).

### Overexpression experiments and RNA sequencing

For overexpression (OE) experiments, cells were treated with doxycycline (250 ng/ml, Sigma-Aldrich, St. Louis, MO, USA) to induce expression of *PRDM10^WT^* or *PRDM10^Cys677Tyr^*. For Illumina-based RNA sequencing specifically, RPTEC/TERT1 WT, RPTEC/TERT1 *FLCN^KO^* and RPTEC/TERT1 *PRDM10^Cys677Tyr^* (upon 120 hours of induction) cell lines were harvested in duplicate and RNA was extracted from the dry cell pellets. Then, samples were prepped using TruSeq Stranded mRNA Library Preparation Kit according to TruSeq Stranded mRNA Sample Preparation Guide. Sequencing was performed on an Illumina HiSeq 4000 (Illumina, San Diego, CA, USA) using run mode SR50. Reads were trimmed using sickle-1.33 (67) and aligned to hg19 using hisat2-2.0.4 (68). The alignments were assigned to genes and exons using featurecount-1.5.0-p3 (69) using the gene annotation provided by the iGenomes resource (70). To compare RNA-sequencing profiles between *PRDM10^Cys677Tyr^* OE and WT RPTECs, the R package edgeR was used (71). Obtained *P*-values were corrected for multiple testing using the Benjamini–Hochberg false discovery rate step-up procedure (72). The volcano plots in Figure 4D and E were generated with VolcaNoseR web app (73). The raw count data are deposited in the Zenodo Repository and are openly available via https://doi.org/10.5281/zenodo.6420633.

## Supplementary Material

Supplemental_Figures_ddac288

Supplemental_tables_final_ddac288
